# ICTV Virus Taxonomy Profile: *Pospiviroidae*

**DOI:** 10.1099/jgv.0.001543

**Published:** 2020-12-17

**Authors:** Francesco Di Serio, Robert A. Owens, Shi-Fang Li, Jaroslav Matoušek, Vicente Pallás, John W. Randles, Teruo Sano, Jacobus Th. J. Verhoeven, Georgios Vidalakis, Ricardo Flores, ICTV Report Consortium

**Affiliations:** ^1^​ Istituto per la Protezione Sostenibile delle Piante, Consiglio Nazionale delle Ricerche, Bari, 70126, Italy; ^2^​ Molecular Plant Pathology Laboratory, US Department of Agriculture, Agricultural Research Service, Beltsville, MD 20705, USA; ^3^​ State Key Laboratory of Biology of Plant Diseases and Insect Pests, Institute of Plant Protection, Chinese Academy of Agricultural Sciences, Beijing, 100193, PR China; ^4^​ Institute of Plant Molecular Biology, Biology Centre of the Czech Academy of Sciences, 37005 České Budějovice, Czech Republic; ^5^​ Instituto de Biología Molecular y Celular de Plantas, Universidad Politécnica de Valencia–Consejo Superior de Investigaciones Científicas, Valencia, 46010, Spain; ^6^​ School of Agriculture, Food and Wine, The University of Adelaide, Waite Campus, Glen Osmond, SA 5064, Australia; ^7^​ Faculty of Agriculture and Life Science, Hirosaki University, Hirosaki 036-8561, Japan; ^8^​ Plant Protection Organization of the Netherlands, Wageningen, 6700 HC, Netherlands; ^9^​ Department of Microbiology and Plant Pathology, University of California, Riverside, CA 92521, USA

**Keywords:** *Pospiviroidae*, ICTV Report, taxonomy

## Abstract

Members of the family *Pospiviroidae* have single-stranded circular RNA genomes that adopt a rod-like or a quasi-rod-like conformation. These genomes contain a central conserved region that is involved in replication in the nucleus through an asymmetric RNA–RNA rolling-circle mechanism. Members of the family *Pospiviroidae* lack the hammerhead ribozymes that are typical of viroids classified in the family *Avsunviroidae*. The family *Pospiviroidae* includes the genera *Apscaviroid*, *Cocadviroid*, *Coleviroid*, *Hostuviroid* and *Pospiviroid*, with >25 species. This is a summary of the ICTV Report on the family *Pospiviroidae*, which is available at ictv.global/report/pospiviroidae.

## Genome

**Fig. 1.**
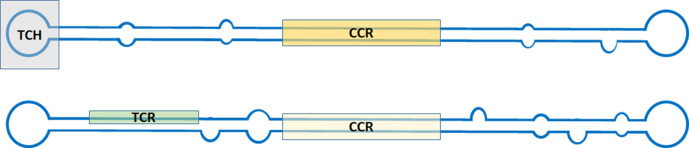
Rod-like structure models for viroids, The positions of the central conserved region (CCR), the terminal conserved region (TCR) and the terminal conserved hairpin (TCH) are indicated by shading. The sequence-specific TCH and TCR elements have never been found together in the same viroid.

Members of the family *Pospiviroidae* have circular single-stranded RNA genomes of a few hundred nucleotides. They may assume rod-like or quasi-rod-like conformations containing a central conserved region (CCR) and a terminal conserved hairpin (TCH) or a terminal conserved region (TCR) (Table 1, Fig. 1) [1–3]. The G+C content is >50 %. The genome of viroids does not encode any proteins.

**Table 1.** Characteristics of members of the family *Pospiviroidae*

Example:	potato spindle tuber viroid (V01465), species *Potato spindle tuber viroid*, genus *Pospiviroid*
Genome	Single-stranded circular RNA of 246–375 nt that adopts a rod-like or quasi-rod-like conformation of minimum free energy and contains typical conserved motifs
Replication	Mediated by nuclear DNA-dependent RNA polymerase II, with oligomeric RNAs of (+) polarity cleaved by a type III RNase and circularized by DNA ligase 1
Translation	Absent
Host range	Plants (dicotyledons and some monocotyledons)
Taxonomy	Several genera including >25 species

## Replication

Replication is nuclear and mediated by DNA-dependent RNA polymerase II, which is redirected to use RNA templates through an asymmetric RNA–RNA rolling-circle mechanism. Circular RNA molecules of (+) polarity (by convention the most abundant strand *in vivo*) are repeatedly transcribed into oligomeric complementary (−) RNAs. Such intermediates serve as templates for generating oligomeric (+) RNAs that are cleaved by a host enzyme of the RNase III class. The termini of the resulting linear monomers are ligated by the host DNA ligase 1 to generate the mature circular viroid RNA [4]. In contrast to members of the family *Avsunviroidae,* the (−) oligomeric RNAs of members of the family *Pospiviroidae* are not cleaved and do not generate the corresponding circular forms.

## Taxonomy

**Fig. 2.**
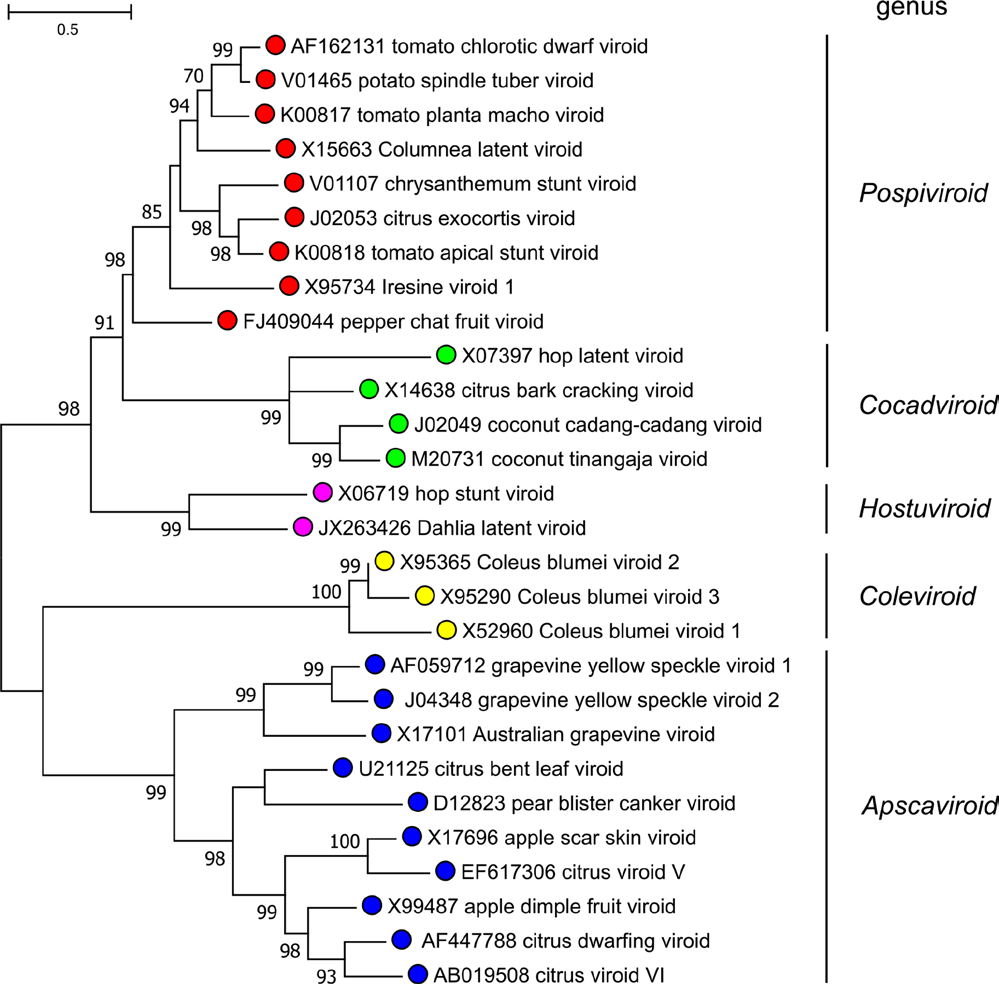
Phylogenetic tree of viroid sequences. Maximum-likelihood analysis was conducted with megax [6]. Nodes are labelled with bootstrap support (1000 replicates) where this was >70%.

Current taxonomy: ictv.global/taxonomy. Demarcation of genera is based upon the type of CCR and the presence of a TCH or TCR (Fig. 1), as well as phylogenetic clustering in trees based upon whole-genome sequences (Fig. 2). Species demarcation criteria include there being <90 % sequence identity and distinct biological properties with respect to other members of the genus [5]. Members of the genus *Pospiviroid*, such as potato spindle tuber viroid, share the same CCR and have a TCR. Most infect herbaceous hosts, mainly solanaceous species. Hostuviroids, such as hop stunt viroid, share the same CCR and have a TCH, except for members of the species *Dahlia latent viroid,* which have a TCR instead of the TCH. Hop stunt viroid has a wide natural host range, while dahlia latent viroid is restricted to *Dahlia* spp. Cocadviroids, such as coconut cadang-cadang viroid, share the same CCR and have a TCH. Some members infect monocotyledons, while others can only infect dicotyledons. Apscaviroids, such as apple scar skin viroid, share the same CCR and have a TCR. Apscaviroids mainly infect woody plants. Coleviroids, such as Coleus blumei viroid 1, share the same CCR and have a TCR or a TCH. The natural host range of coleviroids is restricted to species in the genus *Coleus*.

## Resources

Full ICTV Report on the family *Pospiviroidae*: ictv.global/report/pospiviroidae.
